# Italian Multicenter Real-World Study on the Twelve-Month Effectiveness, Safety, and Retention Rate of Guselkumab in Psoriatic Arthritis Patients

**DOI:** 10.3390/jcm14124111

**Published:** 2025-06-10

**Authors:** Fabiola Atzeni, Cinzia Rotondo, Cesare Siragusano, Addolorata Corrado, Alberto Cauli, Roberto Caporali, Maria Sole Chimenti, Fabrizio Conti, Valentina Picerno, Elisa Gremese, Federica Camarda, Serena Guiducci, Roberta Ramonda, Luca Idolazzi, Angelo Semeraro, Marco Sebastiani, Giovanni Lapadula, Gianfranco Ferraccioli, Florenzo Iannone

**Affiliations:** 1 Rheumatology Unit, Department of Experimental and Internal Medicine, University of Messina, 98168 Messina, Italy; cesaresiragusano@gmail.com; 2 Rheumatology Unit, Department of Medical and Surgical Sciences, Azienda Ospedaliero-Universitaria Policlinico Riuniti di Foggia, Università degli Studi di Foggia, 71122 Foggia, Italy; cinzia.rotondo@gmail.com (C.R.); ada.corrado@unifg.it (A.C.); 3 Rheumatology Unit, Department of Medical Science and Public Health, The University Hospital (AOU), University of Cagliari, 09042 Monserrato, Italy; cauli@unica.it; 4 Rheumatology Unit, Dipartimento di Reumatologia e Scienze Mediche, ASST PINI-CTO, 20122 Milan, Italy; roberto.caporali@unimi.it; 5 Rheumatology Unit, Dipartimento di Scienze Cliniche e di Comunità, University of Milan, 20122 Milan, Italy; 6 Rheumatology, Allergology and Clinical Immunology, Department of Systems Medicine, University of Rome Tor Vergata, 00133 Rome, Italy; maria.sole.chimenti@uniroma2.it; 7 Rheumatology Unit, Department of Clinical Internal, Anesthesiologic and Cardiovascular Sciences, Sapienza University of Rome, 00184 Rome, Italy; fabrizio.conti@uniroma1.it; 8 Rheumatology Department of Lucania, Rheumatology Institute of Lucania (IReL), San Carlo Hospital of Potenza, 75100 Potenza, Italy; valepicerno@tiscali.it; 9 Rheumatology Department of Lucania, Rheumatology Institute of Lucania (IReL), Madonna Delle Grazie Hospital of Matera, 75100 Matera, Italy; 10 Rheumatology and Clinical Immunology, IRCCS Humanitas Research Hospital, 20089 Rozzano, Italy; eli.gremese@gmail.com; 11 Department of Biomedical Sciences, Humanitas University, Pieve Emanuele, 20090 Milan, Italy; 12 Rheumatology Unit, University of Palermo, 90133 Palermo, Italy; federicacamarda@gmail.com; 13 Division of Rheumatology, Department of Experimental and Clinical Medicine, University of Florence, 50139 Florence, Italy; serena.guiducci@unifi.it; 14 Rheumatology Unit, Department of Medicine DIMED, University of Padua, 35131 Padua, Italy; roberta.ramonda@unipd.it; 15 Rheumatology Unit, Department of Medicine, University of Verona, 37134 Verona, Italy; luca.idolazzi@univr.it; 16 Rheumatology Unit, Martina Franca Hospital, 74015 Martina Franca, Italy; angelosemeraro@alice.it; 17 Rheumatology Unit, AUSL Piacenza, University of Parma, 43126 Parma, Italy; marco.sebastiani@unipr.it; 18 GISEA Group, 20100 Milan, Italy; giovanni.lapadula@uniba.it; 19 Department of Medicine, Catholic University Sacred Heart—FPG, 00168 Roma, Italy; gff1990@gmail.com; 20 Rheumatology Unit, DiMePRe-J, University of Bari, 70124 Bari, Italy; florenzo.iannone@uniba.it

**Keywords:** psoriatic arthritis, safety, efficacy, drug survival, Il-23 inhibitor

## Abstract

**Background/Objectives**: Psoriatic arthritis (PsA) is a chronic inflammatory condition that primarily affects the musculoskeletal system and skin. While biologic and targeted synthetic DMARDs have improved treatment, many patients still fail to achieve remission. Combining conventional synthetic disease modifying anti-rheumatic drugs (csDMARDs) with biologic (b) DMARDs or targeted synthetic (ts) DMARDs shows no added benefit over monotherapy with IL-17, IL-23 inhibitors, or JAK inhibitors, unlike TNFi, which benefit from csDMARD co-administration. Guselkumab (GUS) and risankizumab (RKZ) target IL-23 with high specificity. RCTs (DISCOVER 1 and 2, COSMOS) have confirmed GUS efficacy regardless of methotrexate (MTX) use, though liver toxicity was higher with MTX. Real-world data on GUS remain limited, with gaps in understanding its long-term effectiveness and drug survival. The aim of this study is to assess the following three points within a multicenter Italian real-life cohort of PsA patients treated with guselkumab (GUS) and followed for 12 months: (1) effectiveness and safety of GUS; (2) drug retention rate (DRR) and reasons for discontinuation; (3) impact of comorbidities on achieving minimal disease activity (MDA). **Methods**: This study utilized data from the GISEA registry, which includes centers in different parts of Italy (north, center, south, and islands), and included patients aged 18 and older diagnosed with PsA according to the CASPAR criteria. **Results:** Data on 170 PsA patients treated with GUS were collected. In the first 6 months, a prompt mean percentage improvement in all clinimetric indexes was observed compared to the baseline. At 6-month follow-up, ACR 20 was reached by 60% of patients, ACR 50 by 30%, ACR 70 by 15%, MDA by 28%, and DAPSA < 14 by 50% of patients in the overall group. Significant differences were found in the rate of ACR 50 in the bDMARD-naive group (50%) compared to one bDMARDs non-responder (NR) (8%) (*p* = 0.021). At 12-month follow-up, a notable gap was observed in the rate of patients reaching MDA between bDMARD-naive (60%) and one bDMARDs NR (22%) (*p* = 0.035) and between bDMARD-naive (60%) and ≥2 bDMARDs NRs (22%) (*p* = 0.024). By using multivariate binary logistic analysis, the predictors of reaching MDA at 12-month follow-up were naive bDMARDs (OR: 7.9, 95% CI: 1.3–44.8, *p* = 0.019) and a higher value of pGA at baseline (OR: 1.1, 95% CI: 1–1.5; *p* = 0.046). The presence of comorbidities, including fibromyalgia and obesity, did not seem to affect the reaching of MDA. At 12-month follow-up, the GUS retention rate was 76%, with a mean survival time of 10.5 months ± 0.2 (95% CI: 10–10.9). No significant differences in GUS survival time were found among bDMARD-naive, one bDMARDs NR, and ≥2 bDMARDs NR patients (in the latter, regardless of the previous mechanism of action: TNFi or other mechanism), as well as between patients treated with GUS in monotherapy and those treated in combination with csDMARDs. A low rate (17%) of discontinuation was found due to both primary NR and secondary NR. The high safety of GUS was recorded. In fact, discontinuation due to adverse events (all definable as minor) was observed in just 4% of patients. By using COX regression multivariate analysis, the factors associated with higher GUS discontinuation risk were a more severe baseline PASI (HR: 1.05, 95% CI: 1–1.1, *p* = 0.038) and higher baseline ESR (HR:1.06, 95% CI: 1–1.03, *p* = 0.05). **Conclusions:** Good performance of GUS was observed in both biologic-naive patients and those with failure of previous bDMARDs (regardless of the mechanism of action of the previous drug: TNFi or non-TNFi), presenting good persistence in therapy even when used as a third mechanism of action. Its high safety profile allows the use of GUS even in particularly complex patients.

## 1. Introduction

Psoriatic arthritis (PsA) is a chronic inflammatory condition affecting both the musculoskeletal system and the skin, primarily involving peripheral joints, entheses, the axial skeleton, and various skin areas [1,2]. In recent years, various biologic and targeted synthetic disease-modifying rheumatic drugs (DMARDs) have demonstrated efficacy in treating PsA by transforming the disease course and improving patients’ functional status [3]. However, as a significant number of patients still did not reach remission or low disease activity, the need for more effective treatment approaches is growing [3,4,5]. In this context, combining conventional synthetic (cs) DMARDs with biologic (bDMARDs) or targeted synthetic DMARDs (tsDMARDs) does not appear to offer superior effectiveness compared to monotherapy with anti-Interleukin 17 (IL-17), anti-Interleukin 23 (IL-23), and Janus kinase inhibitors (JAKi) [3]. This trend contrasts with tumor necrosis factor inhibitors (TNFi), where co-administration with a csDMARD has been shown to enhance treatment persistence [6]. As the pivotal role of IL-23 in PsA pathogenesis became clearer, two monoclonal antibodies (guselkumab (GUS) and risankizumab (RKZ)), targeting the p19 subunit with high affinity and specificity, were introduced into the treatment arsenal [7,8,9]. DISCOVER 1 was the first double-blind, phase III randomized clinical trial (RCT) to evaluate the efficacy and safety of GUS in patients with active PsA who had failed one or two prior bDMARDs [8]. The primary endpoint of achieving an ACR20 response at week 24 was met, regardless of concurrent methotrexate (MTX) use [10]. DISCOVER 2, a similarly designed RCT, also supported these findings, assessing the efficacy, safety, and structural impact of GUS in active PsA patients who were bDMARD-naive and stratified by csDMARD use [10]. COSMOS, a phase IIIb RCT with PsA patients showing inadequate response to TNFi, randomized patients to receive either GUS 100 mg every 8 weeks or a placebo, showing consistent subgroup results based on concurrent MTX use [11]. No significant differences in safety were observed, though higher rates of liver toxicity were noted in patients using MTX alongside GUS, corroborated by the open-label extension (OLE) of the DISCOVER 2 trials [12]. Few studies have explored the real-world effectiveness and safety of GUS, both within Italian cohorts and internationally, though these studies have generally covered only a limited observational period [13,14,15,16,17,18]. Furthermore, the effects of prior lines of biologic bDMARDs, comorbidities, and specific clinical features on achieving clinical remission in GUS drug survival remain largely unexamined.

Nowadays, the importance of real-world data is increasing more and more, due to some RTC limitations such as strict inclusion criteria and short follow-up. Furthermore, real-life data on drug efficacy and safety are increasingly important for integrating RCT data, identifying subgroups of patients who respond best to therapy, improving therapeutic strategies, and providing regulatory authorities with important data for informed decisions. The Italian Health system is based on principles of universality, equality, and equity, which differentiate it from health systems in other nations. For about 5 years, for spending review policies, although accessibility to care is always guaranteed, many Italian regions require the first-line use of lower-cost drugs.

This study aimed to assess the following within a multicenter Italian real-life cohort of PsA patients treated with GUS and followed for 12 months: (1) effectiveness and safety; (2) drug retention rate (DRR) and reasons for discontinuation; and (3) the impact of comorbidities and previous bDMARD treatment lines on achieving minimal disease activity (MDA).

## 2. Materials and Methods

This study utilized data from the GISEA registry, which includes centers in different parts of Italy (north, center, south, and islands), and included patients aged 18 and older diagnosed with PsA according to the CASPAR criteria [19]. Axial involvement was assessed using the ASAS criteria for axial spondyloarthritis [20]. Patients were either biologic-naive or had previously shown inadequate responses to biologics, beginning GUS treatment between January 2022 and 2023. This study was approved by the Ethics Review Board at the Policlinico of Bari (GISEA Registry-protocol no. 598/2011) and complied with the Declaration of Helsinki. Written informed consent was obtained from all patients for data use in publications, with guarantees of confidentiality. At the initiation of GUS treatment, several demographic and disease-related characteristics were recorded. These included age, gender, BMI, clinical presentation (axial, peripheral, or mixed), other clinical manifestations (such as enthesitis, dactylitis, skin or nail involvement, and extra-articular symptoms), disease duration, age at diagnosis, previous or concurrent treatments, and current medications (including csDMARDs, NSAIDs, glucocorticosteroids, or previous biologic agents). Given the expected variation in treatment retention and response between biologic-naive patients and those with prior biologic bDMARD exposure, information was gathered on first-line treatment and subsequent lines before GUS initiation. Patient-reported outcomes (PROs) included the Visual Analogue Scale for pain (VAS pain, 0–100), patient global assessment (pGA, 0–100), physician global assessment (phGA, 0–100), health assessment questionnaire disability index (HAQ-DI, 0–3), and Bath Ankylosing Spondylitis Disease Activity Index (BASDAI, 0–10). Clinical evaluations consisted in the Psoriasis Area Severity Index (PASI, 0–72), assessment of psoriatic onychopathy (yes/no), tender joint count (TJC, 0–68), and swollen joint count (SJC, 0–66). Patients with peripheral involvement were categorized according to Disease Activity Index for Psoriatic Arthritis (DAPSA) scores: remission (DAPSA ≤ 4), low disease activity (DAPSA > 4 and ≤14), moderate disease activity (DAPSA > 14 and ≤28), or high disease activity (DAPSA > 28) [21]. For patients with axial involvement, the Ankylosing Spondylitis Disease Activity Score (ASDAS, CRP-based) was used to describe four levels of disease activity: inactive disease (ID) (<1.3), low disease activity (LDA) (1.3 to <2.1), high disease activity (2.1 to 3.5), and very high disease activity (>3.5) [22,23]. Enthesitis was measured via the Leeds Enthesitis Index (LEI, 0–6), and dactylitis was assessed by the number of affected digits (0–20). Minimal disease activity (MDA) or very low disease activity (VLDA) was achieved if patients met five (MDA) or seven (VLDA) of the following outcomes: TJC68 ≤ 1, SJC66 ≤ 1, LEI ≤ 1, PASI score ≤ 1, VAS pain ≤ 15 mm, VAS pGA ≤ 20 mm, and HAQ-DI score ≤ 0.5 [24]. The proportions of patients achieving DAPSA remission/LDA, MDA, VLDA, and ASDAS-CRP ID/LDA were tracked over time. Patient assessments occurred at baseline, at 6 months, and 12 months of GUS treatment. Additionally, any comorbidities were documented and defined as medical conditions distinct from the primary PsA diagnosis for which the patients were included in this study. The inclusion criteria were age over 18 years, fulfilment of the CASPAR classification criteria for PsA [19], and treatment with GUS in monotherapy or stable combination treatment with GUS and csDMARDs (methotrexate, leflunomide). Patients younger than 18 years of age who did not meet CASPAR criteria and who had positive rheumatoid factors were excluded. The failure of previous bDMARDs was defined by DAPSA and in case of skin involvement by PASI.

### Statistical Analysis

The results are expressed as mean ± S.D. (standard deviation) and percentage. The normal distribution was assessed using the Shapiro–Wilk test. Comparisons between study groups of PsA patients were evaluated by Student’s *t*-test or Mann–Whitney U-test, as appropriate. Comparisons of continuous variables among the groups of patients in different GUS lines of treatment were realized with analysis of variance (ANOVA). The changes in continuous variables at the different time points of this study were analyzed by ANOVA for repeated measures, followed by the Bonferroni test in case of comparisons between groups of patients. The differences between categorical variables were assessed by Pearson chi-squared or Fisher’s exact test, as appropriate, followed by Bonferroni adjustment in the case of multiple comparisons. Univariate and multivariate binary logistic regression analyses were used to evaluate the factors associated with reaching MDA at the 12th month of follow-up. The estimation of drug survival was realized via Kaplan–Meier estimate, followed by the log-rank (Mantel–Cox) test in case of comparisons between different groups of patients. The risk of drug discontinuation was evaluated using the Cox regression model and presented as hazard ratio (HR) and 95% confidence interval (CI). Missing data ranged between 5% and 12%. Therefore, data imputation was not performed. Statistical significance was set at *p* ≤ 0.05. Statistical analysis was assessed using IBM SPSS Statistics 26.

## 3. Results

### 3.1. Demographic and Clinical Characteristics of the Patients

Data on 170 PsA patients treated with GUS were collected from January 2022 to May 2024 in 12 Italian centers. Demographic, clinical, and disease characteristics are reported in Table 1. The patients’ mean age at the start of GUS therapy was 55.7 years (±11.4). In total, 35% of patients were female, and the disease duration of PsA was 12.1 years (±5.1). In total, 100 (59%) patients were treated with GUS in monotherapy, while 70 (41%) patients were treated with GUS in combination with csDMARDs, including leflunomide, methotrexate, sulfasalazine, hydroxychloroquine, or cyclosporine A. Forty-one (24%) patients received concomitant oral glucocorticoids. Of note, 26 (15%) patients were bDMARD-naive, 26 (15%) were non-responders (NRs) to one previous bDMARDs (54% TNFi and 46% other mechanisms of action), and 118 (70%) patients were NR to two or more bDMARDs (20% TNFi, 5% other mechanisms of action, and 75% both TNFi and other mechanisms of action) (Table 1 and Table 2).

Considering patients classified into groups according to GUS treatment lines, the patients’ ≥2 bDMARDs NRs were significantly older (57.8 years ± 10.2, *p* = 0.016) and affected by longer disease duration (13.7 years ± 9.2, *p* = 0.006), compared to both bDMARD-naive patients (51 years ± 15.2 and 6.4 years ± 6.8, respectively) and one bDMARDs NR (50.3 years ± 9.4 and 10.8 years ± 8.8, respectively). No other significant differences were found among these groups of patients at baseline (Table 2).

### 3.2. Clinical Response to Guselkumab and Predictors of Efficacy

After 6 months of GUS treatment, a significant improvement in all clinimetric indexes was found, including DAPSA, tender joint count, swollen joint count, VAS pain, LEI, pGA, phGA, PASI, and HAQ (Table 2). Of note, in the first six months, a prompt mean percentage improvement of 42% in DAPSA, 53% in tender joint counts, 72% in the swollen joint count, 54% in LEI, 42% in phGA, and 83% in PASI was observed compared to the baseline. All data are shown in Table 2. This positive trend of improvement in clinimetric indexes was also observed during the follow-up at 12 months (Table 2).

Even though an improvement in all clinimetric evaluations at 6 and 12 months was observed, no significant differences were found among groups of patients in different lines of bDMARD treatments (Table 3). However, a significant improvement was observed in pGA at 12 months in the bDMARD-naive patient group compared to one bDMARDs NR (*p* = 0.0001) and ≥2 bDMARDs NRs (*p* = 0.0011) and between ≥2 bDMARDs NRs and 1 bDMARDs NR (*p* = 0.017) (Table 3).

**Figure 1:**
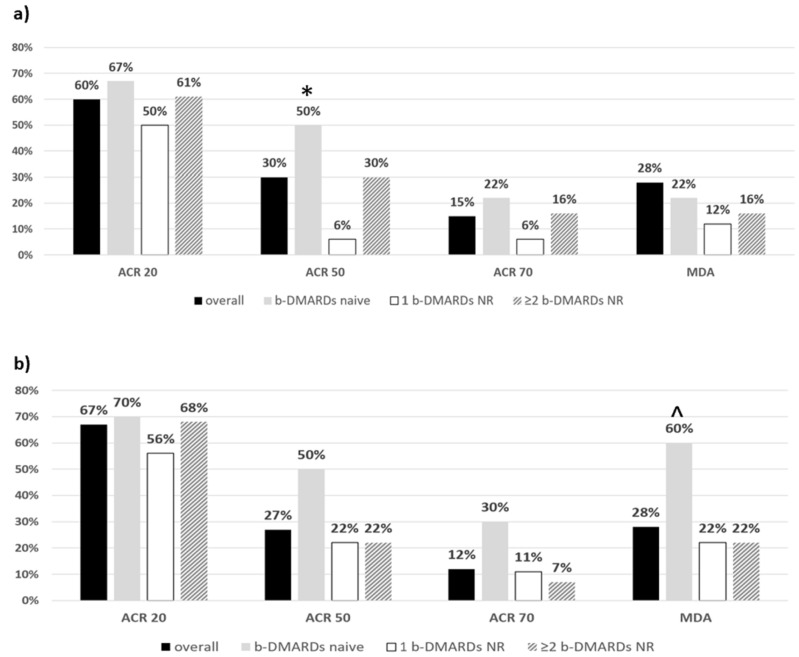
Clinical response to guselkumab treatment according to ACR 20, ACR 50, ACR 70, and MDA in overall patients and in the group stratified for guselkumab treatment lines: (**a**) at 6 months; (**b**) at 12 months. Statistical analysis: Pearson chi-squared test followed by Bonferroni test. Abbreviation: DMARDs: disease-modifying antirheumatic drugs; MDA: minimal disease activity. * bDMARD-naive vs. 1 bDMARDs NR, *p* = 0.021. ^ bDMARD-naive vs. 1 bDMARDs NR, *p* = 0.035; bDMARD-naive vs. 2 or more bDMARDs NR, *p* = 0.024.

At the 6-month follow-up, the ACR 20 was reached by 60% of the patients in this study, ACR 50 by 30%, ACR 70 by 15%, MDA by 28%, and DAPSA < 14 by 50% of patients in the overall group. In particular, considering the bDMARD treatment lines, significant differences were found in the rate of ACR 50 in the bDMARD-naive group (50%) compared to one bDMARDs NR (8%) (*p* = 0.021) (Figure 1a).

At the 12-month follow-up, the ACR 20 was observed in 67% of patients, ACR 50 in 27%, ACR 70 in 12%, MDA in 28%, and DAPSA < 14 in 70% of patients in the overall group. Of note, a notable gap was observed in the rate of patients reaching MDA between bDMARD-naive (60%) and one bDMARDs NR (22%) (*p* = 0.035), and between bDMARD-naive (60%) and two or more bDMARDs NRs (22%) (*p* = 0.024) (Figure 1b).

By univariate binary logistic analysis, the predictors of reaching MDA at 12-month follow-up were bDMARD-naive (OR: 7.1, 95%CI 1.4–34.2; *p* = 0.014), higher baseline value of pGA (OR: 1.3, 95%CI 1–1.7; *p* = 0.048), and more severe PASI at baseline (OR 1.1, 95% CI: 1–1.4; *p* = 0.031). By multivariate binary logistic analysis, the predictors of reaching MDA at 12-month follow-up were bDMARD-naive (OR: 7.9, 95% CI: 1.3–44.8, *p* = 0.019) and higher baseline value of pGA at baseline (OR: 1.1, 95% CI: 1–1.5; *p* = 0.046) (Table 4). The presence of comorbidities, including fibromyalgia and obesity, and the combination treatment with csDMARDs did not seem to affect the reaching of MDA.

### 3.3. Retention Rate of Guselkumab at 12 Months

**Figure 2:**
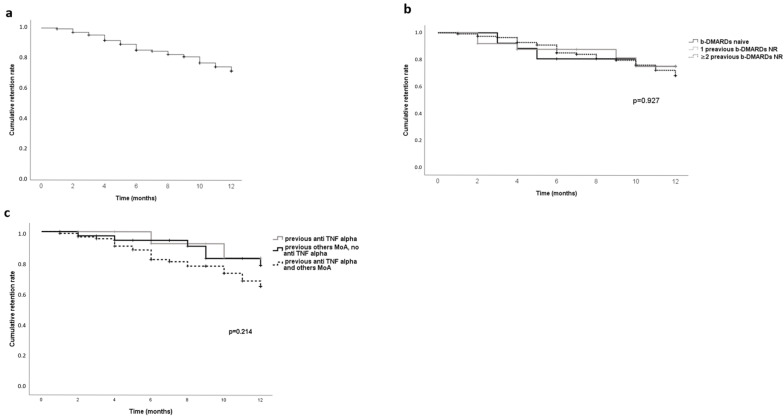
Kaplan–Meier method followed by the log-rank (Mantel–Cox) test. Guselkumab drug survival in psoriatic arthritis patients; (**a**) overall; (**b**) stratification for guselkumab treatment lines; (**c**) stratification for previous mechanism of action used before guselkumab. Abbreviation: DMARDs: disease-modifying antirheumatic drugs; MoA: mechanisms of action; TNF: tumor necrosis factor.

At the 12th month of follow-up, the GUS retention rate was 76% with a mean survival time (MST) of 10.5 months ± 0.2 (95% CI: 10–10.9) (Figure 2a).

Stratifying patients in groups according to GUS line treatment, no significant differences (*p* = 0.912) were found in survival time among the groups. The retention rates of each study group were 76.9% in the bDMARD-naive group (with MST: 10.3 months ± 0.6, 95% CI: 9.1–11.5), 81% in the one bDMARDs NR group (with MST: 10.5 months ± 0.6, 95% CI: 9.3–11.7), and 75% in the ≥2 bDMARDs NRs group (with MST: 10.5 months ± 0.2, 95% CI: 10–11) (Figure 2b). Of note, considering patients in ≥2 bDMARDs NRs, no differences were observed in MST compared to the group of patients previously treated with TNFi in monotherapy (11.3 months ± 0.4), those treated with other mechanisms of action (except TNFi) (11.1months ± 0.4), and those previously treated with TNFi and then with other mechanisms of action (10.6 months ± 0.3) (*p* = 0.214) (Figure 2c).

Considering the group of patients treated with GUS in monotherapy and those treated in combination with csDMARDs, no differences were observed in survival time (*p* = 0.096). The retention rates were 72% (with MST: 10.2 months ± 0.3, 95% CI: 9.5–10.8) and 81.4% (with MST: 10.9 months ± 0.3, 95% CI: 10.2–11.5), respectively.

Discontinuation due to primary non-response was observed in 17% of patients and in another 17% of patients for secondary non-response. Discontinuation for adverse events (AEs) (all defined as minor AEs) was observed in 4% of patients.

Specifically considering the reason for drug discontinuation, the GUS survival time for the primary non-response was 5.4 months ± 0.5 (95% CI: 4.5–6.3), the MST for the secondary non-response was 10.5 months ± 0.4 (95% CI: 9.6–11.4), and the MST for adverse events was 4.6 months ± 0.6 (95% CI: 3.3–5.9).

The Cox proportional hazard model was used to assess the factors associated with GUS discontinuation. By using univariate analysis, poor GUS drug retention was related to high baseline DAPSA (HR: 1.02, 95% CI: 1.0–1.05, *p* = 0.011), more severe cutaneous psoriasis scored by PASI (HR: 1.06, 95% CI: 1.0–1.17, *p* = 0.017), and higher baseline erythrocyte sedimentation rate (ESR) (HR: 1.02, 95% CI: 1.0–1.03, *p* = 0.001). By using multivariate analysis, the factors associated with higher GUS discontinuation risk were a more severe baseline PASI (HR: 1.05, 95% CI: 1–1.1, *p* = 0.038) and higher baseline ESR (HR:1.06, 95% CI: 1–1.03, *p* = 0.05).

## 4. Discussion

In a scenario of real-life experience data on GUS, this is the first study including a larger cohort of 170 patients derived from the Italian multicenter registry (GISEA), in which a good efficacy and safety profile of GUS in PsA patients was demonstrated at 6 and 12 months of follow-up. Indeed, previously published studies had limited cohorts of patients: 114 patients in Mease et al. [16], 111 patients in Ruscitti et al. [14], and 24 patients in Pantano et al. [13]. Furthermore, for the first time, real-life data are published that include patients from all parts of Italy (south, center, and north, including the islands). In line with prior real-world studies and clinical trials [13,14,15,16,17,18], GUS effectiveness is underlined by prompt (at 6th months) and continuous (at 12th months) improvements in all clinimetric parameters and clinical multi-domains such as DAPSA, TJC, SJC, VAS pain, LEI, phGA, pGA, PASI, and HAQ. These improvements were present in all lines of GUS treatment, reconfirming what was previously demonstrated [8,10,11,12,13,16].

Similarly to other published studies, our cohort of patients was also characterized by a long duration of disease, and predominantly by previous failures of bDMARDs due to some national restrictions in the first-line prescription of non-TNFi. Although in Italy there are some prescribing limitations, preferring the use of biosimilars in the first line of bDMARDs, this condition did not seem to limit the accessibility of care in Italy compared to other populations. In fact, the published rate of b/tsDMARD-experienced patients in real-world studies on GUS from the data was similar in different countries: 92% in the CorEvitas registry (United State) and 87% in the Greek registry [25].

MDA was reached by 28% of patients at both the 6th and 12th months of the study period, a slightly higher percentage than that observed by Mease (21.5% at 6 months) [16], confirming the prompt clinical response to GUS. In particular, considering the different study subgroups, MDA was reached at 12 months by bDMARD-naive patients (60%) (of which only approximately 40% was in combined treatment with csDMARDs). This could be due to the low sample size and should not be indicative of particular clusters of patients in which GUS is more effective. But in any case, this could indicate the generally better performance of bDMARDs as the first line of treatment and in the earliest phases of the disease, in our study, regardless of the concomitant use of csDMARDs, in line with all registered studies on bDMARDs [8]. Furthermore, considering the complexity of psoriatic disease with different disease domains, and the wide spectrum of clinical manifestations included in the MDA (articular, cutaneous, enthesis domains, and patient-reported outcomes including HAQ), it is likely that achieving the MDA is very complex and may require longer times, such as 12 months rather than 6 months. This does not mean that, if we consider the different disease domains individually, we may not see faster responses for some of them.

Regarding the possibility of reaching MDA at 12-month follow-up, predictive factors are considered to be naive to bDMARDs and a higher baseline value of pGA, demonstrating the benefit of GUS on the patient’s perception of disease activity. This target does not appear to be influenced by comorbidities, such as fibromyalgia and obesity, in line with previous studies [14]. This last finding should not be underestimated. In fact, PsA is associated with a higher prevalence of comorbidities such as metabolic syndrome, depression, fibromyalgia, and so on, compared to the general population [26]. Moreover, the presence of comorbidities often complicates both therapeutic management and response to drugs [27,28,29,30,31], mainly TNFi [32], and there are no specific studies on the influence of fibromyalgia on the efficacy of bDMARDs with mechanisms of action other than TNFi. In the post-hoc analysis of the DISCOVER 1 and 2 trials, patients with fibromyalgia also experienced an improvement in pain, although slower than patients without fibromyalgia but still significant compared to patients in the placebo group. A potential effect of GUS in improving pain in PsA patients with fibromyalgia was also hypothesized [33,34]. The results of this study regarding the absence of the influence of comorbidities in reaching MDA, in particular fibromyalgia, add an important piece to the hypotheses previously published, although it cannot be ruled out that the sample of patients is too small, so further studies on larger samples could confirm or deny these findings.

Furthermore, the results of this study could strongly impact therapeutic strategies, considering the greater possibility of reaching MDA in patients with higher pGA and especially in first-line treatment with bDMARDs. These findings can support therapeutic choices and also suggestions provided in the recommendations of the most important scientific societies, which could also consider, among other clinical parameters, the PROs as drivers of choice between the different therapeutic possibilities available. In fact, the current main scientific societies’ recommendations for PsA treatment (GRAPPA [35] and EULAR [36]), although considering the different individual clinical domains of PsA as a driver for choosing different mechanisms of action, do not give importance to the patient’s perception of the disease. Our results about pGA and the efficacy and safety profile of GUS in patients with comorbidities could provide a further driver of choice in future drafts of the recommendations.

This study also obtained a higher percentage of patients achieving a DAPSA < 14 (50%) at 6-month follow-up compared to 39.6% observed by Ruscitti et al. [14], although in both studies, patients had moderate disease activity at baseline, assessed by DAPSA. This study evaluated the GUS effectiveness based on ACR 20, ACR 50, and ACR 70 for the first time in clinical practice, and it reached 6 months by 60%, 30%, and 15% of patients, respectively. This was described in randomized clinical trials but never reported in real-world studies, and it is perfectly in line with findings reported in the DISCOVER 1 study: ACR 20 was reached in 52% of patients, ACR50 was reached in 30%, and ACR 70 was reached in 12% [10]. This good ACR response is also maintained at 12 months. In particular, at 6-month follow-up, ACR 50 is largely (50%) reached in the bDMARD-naive group compared to 1 bDMARDs NR (8%) (*p* = 0.021), echoing a more rapid performance of all bDMARDs in naive patients.

A good (76%) retention rate at 12 months was observed, without significant differences among groups of patients in different lines of bDMARD treatment (naive: 76%, 1 bDMARDs NR: 81%, and ≥2 bDMARDs NRs: 75%), and between GUS monotherapy (72%) and GUS in combination with csDMARDs (81%), this small difference could depend mainly on selection bias. This result emphasized GUS versatility in different clinical settings. These good retention rates in both overall and distinct subsets of patients reinforce the previous rates reported, in particular at 12 months in COSMOS (88%) [11] and DISCOVER 1 (94%) [10] and at 18 months of observation (67%) in real-life studies [14].

An absolute novelty for both real-life studies and clinical trials are the data on the GUS retention rate at 12-month follow-up among the group of patients who failed ≥2 bDMARDs split on the basis of the mechanism of action of previous bDMARDs, such as just TNFi or other mechanisms of actions except TNFi (or both TNFi and another mechanism of actions beyond the TNFi). These findings underlined the efficacy in terms of good persistence in therapy in patients who used GUS as a second mechanism of action and also in multi-failure patients treated with drugs with different mechanisms of action. In general but especially in Italy where cost containment in the health system is in force with the indication for first-line use of biosimilar bDMARDs, efficacy data of GUS in other lines of treatment, regardless of the mechanism of action used previously, can enrich national therapeutic algorithms and could impact the PSA treatment recommendation updates of principal scientific societies.

Data from this study confirm the high safety profile of GUS, which was discontinued in only 4% of patients for AEs, all of them of low clinical relevance, as reported by Ruscitti et al. [15] (4.5%). Regarding the discontinuation of the drug due to both primary non-response and secondary ineffectiveness, a low percentage of patients (17%) was recorded, consistent with clinical trial findings. Of note, in this study, the principal predictive factor for discontinuation is a more severe PASI at baseline, demonstrating how the presence of severe skin psoriasis, which causes serious social and psychological problems for patients, significantly influences therapeutic management. Regarding comorbidities, it is important to underline the high safety profile of GUS, despite the presence of patients with a history of major cardiovascular events (10%), diabetes (15%), and liver disease (23%).

All in all, considering the main findings of this study, it would be useful, in clinical practice, to prioritize GUS for biologic-naïve patients with high-baseline pGA. Although, to significantly impact recommendations in this sense, head-to-head drug superiority studies would be needed.

Limitations: due to the retrospective design of this study, some limitations can be described. First, the low sample size does not allow absolute validation of the results. Second, the clinical heterogeneity of the enrolled patients can influence the real evaluation of disease activity, with a possible impact on effectiveness results. Third, subgroup analyses (e.g., MDA predictors) may be underpowered due to the lack of sample size calculations. Finally, a limited (12-month) treatment period is described in this study. Studies with observation periods of 18 months have already been published, even if they have a smaller sample size.

## 5. Conclusions

Clinical practice shows a good performance of GUS in both biologic-naive patients and those with failure of previous bDMARDs (regardless of the mechanism of action—TNFi or non-TNFi—of the previous drug), presenting good persistence in therapy even when used as a third mechanism of action. Efficacy data and the high safety profile allow GUS use in particularly critical types of patients, such as those with comorbidities. Further data obtained from larger samples, longer observation periods, and possibly on genetic variables would be useful to confirm and expand the validity of these findings and the true effectiveness of IL-23 inhibition in managing PsA in all clinical domains.

## Figures and Tables

**Table 1:** Demographic characteristics of the whole group of patients with psoriatic arthritis treated with guselkumab.

Baseline Characteristics	Patients N = 170
Age (years)	55.7 ± 11.4
Female n (%)	60 (35%)
Disease duration (years)	12.1 ± 9.1
BMI (kg/m^2^)	27.1 ± 5.1
Smokers n (%)	57 (34%)
Family history of psoriasis n (%)	58 (34%)
Enthesitis n (%)	96 (56%)
Dactylitis n (%)	35 (21%)
Concomitant glucocorticoids n (%)	41 (24%)
Concomitant csDMARDs n (%)	70 (41%)
Guselkumab treatment lines	
Naive	26 (15%)
1 previous bDMARD	26 (15%)
Anti-TNFα	14 (54%)
other mechanisms of action no anti-TNFα	12 (46%)
≥2 previous bDMARDs	118 (70%)
Anti-TNFα	23 (20%)
Other mechanisms of action no anti-TNFα	6 (5%)
Both anti-TNFα and other mechanisms of action	89 (75%)
Comorbidities and extra-articular manifestations	
Uveitis	1 (0.6%)
Cutaneous psoriasis n (%)	142 (84%)
Major cardiovascular events	17 (10%)
Dyslipidemia	59 (35%)
Diabetes	26 (15%)
Hepatopathy	39 (23%)
Metabolic syndrome	34 (20%)
Osteoporosis	24 (14%)
Fibromyalgia	62 (36%)

**Table 2:** Changes in clinical characteristics in the overall group at baseline, 6th month, and 12th month.

	Baseline	6th Month	12th Month	*p*-Value
DAPSA (95%CI)	22.4 ± 11.6 (19.3–25.5)	13 ± 8.8 (10.6–15.3)	11 ± 8.3 (8.8–13.3)	0.0001 *
TJC (95%CI)	7.2 ± 6.8 (5.4–9.0)	3.4 ± 4.1 (2.4–4.5)	2.9 ± 4.1 (1.8–4.0)	0.0001 *
SJC (95%CI)	2.5 ± 3.4 (0.4–1.1)	0.7 ± 1.3 (0.4–1.1)	0.4 ± 1.2 (0.1–0.8)	0.0001 *
VAS pain (95%CI)	6.5 ± 2.2 (5.9–7.1)	4.6 ± 2.5 (3.9–5.2)	4.1 ± 2.4 (3.5–4.7)	0.0001 *
LEI (95%CI)	1.1 ± 1.6 (0.6–1.7)	0.5± 0.9 (0.2–0.8)	0.6 ± 1.6 (0.0–1.1)	0.019 °
pGA (95%CI)	6.3 ± 2.1 (5.7–6.9)	4.3 ± 2.4 (3.7–5.0)	4.2 ± 2.3 (3.6–4.8)	0.0001 *
phGA (95%CI)	5 ± 2.3 (4.4–5.6)	2.9 ± 2 (2.4–3.4)	2.2 ± 2 (1.6–2.7)	0.0001 ^§^
ESR mm/h (95%CI)	20.2 ± 14.1 (16.1–24.2)	17.6 ± 12.4 (14.0–21.2)	19.1 ± 16.5 (14.3–23.8)	0.364
CRP mg/L (95%CI)	3.7 ± 4.8 (2.4–5.1)	2.7 ± 3.6 (1.6–3.7)	2.7 ± 5.2 (1.3–4.2)	0.152
PASI (95%CI)	3.6 ± 4.6 (2.3–4.9)	0.6 ± 1.2 (0.3–1.0)	0.4 ± 0.8 (0.1–0.6)	0.0001 *
HAQ (95%CI)	0.6 ± 0.4 (0.4–0.8)	0.5 ± 0.4 (0.3–0.7)	0.4 ± 0.4 (0.2–0.5)	0.002 ^

Abbreviation: CRP: C reactive protein; DAPSA: disease activity in psoriatic arthritis score; ESR: erythrocytes sedimentation rate; HAQ: health assessment questionnaire; LEI: Leeds Enthesitis Index; PASI: Psoriasis Area and Severity Index; pGA: patient global assessment; phGA: physician global assessment; SJC: swollen joint count; TJC: tender joint count; VAS: Visual Analogue Scale. ° baseline vs. 6th month *p* = 0.007. * baseline vs. 6th month *p* = 0.0001 and baseline vs. 12th month *p* = 0.0001. ^§^ baseline vs. 6th month *p* = 0.0001, baseline vs. 12th month *p* = 0.0001; 6th month vs. 12th month *p* = 0.0001. ^ baseline vs. 12th month *p* = 0.011; 6th month vs. 12th month *p* = 0.023.

**Table 3:** Comparison of clinical characteristics among patients in different guselkumab lines of treatment at baseline, 6th month, and 12th month.

	bDMARD-Naive (n = 26)			bDMARDs Non-Responder						*p*-Value
				1 bDMARDs NR (n = 26)			≥2 bDMARDs NR (n = 118)			
	Baseline			Baseline			Baseline			
Female n (%)	16 (61)			18 (69)			76 (69)			0.839
Age (years) (95% CI)	51 ± 15.2 (44.9–57.2)			50.3 ± 9.4 (46.5–54.2)			57.8 ± 10.2 (56–59.7)			0.001 °
Disease duration (years) (95% CI)	6.4 ± 6.8 (3.6–9.3)			10.8 ± 8.8 (7.1–14.4)			13.7 ± 9.2 (11.9–15.3)			0.001 ^
BMI (kg/m^2^)	28.4 ± 5.6 (25.9–30.9)			27.3 ± 6.2 (24.6–29.9)			27.1 ± 4.7 (26.2–28)			0.576
Smokers n (%)	7 (27)			6 (24)			44 (38)			0.305
Concomitant oral GC n (%)	3 (11)			7 (28)			31 (28)			0.218
Concomitant csDMARDs n (%)	11 (42)			7 (27)			52 (41)			0.272
**Time Points**	**Baseline**	**6th Month**	**12th Month**	**Baseline**	**6th Month**	**12th Month**	**Baseline**	**6th Month**	**12th Month**	***p*-Value**
DAPSA (95% CI)	22.3 ± 16.3 (15.8–28.8)	7.8 ± 7.3 (5.4–10.2)	6.6 ± 9.6 (0.6–14.7)	22.2 ± 11.4 (15.0–29.4)	12 ± 7.2 (8.6–15.4)	11.3 ± 10.1 (3.5–19.1)	22.5 ± 10.7 (20.1–24.9)	14.4 ± 9.2 (8.9–19.9)	12 ± 7.6 (9.9–14.6)	0.259
TJC (95% CI)	7.6 ± 10.4 (2.7–12.5)	3.5 ± 5.4 (0.7–6.3)	3 ± 6.5 (0.6–5.4)	7 ± 7.2 (3.5–10.5)	3.1 ± 2.9 (1.9–4.5)	3.2 ± 5.2 (0.8–7.2)	7.2 ± 6.8 (6.2–8.2)	3.5 ± 4.1 (1.1–5.9)	2.8 ± 3.2 (1.4–4.2)	0.974
SJC (95% CI)	3 ± 3.3 (0.9–5.1)	0.2 ± 0.8 (0.0–0.6)	0.0 ± 0.0 (0.0–0.0)	2.3 ± 2.5 (0.6–4)	0.5 ± 0.7 (0.1–1.2)	0.3 ± 0.5 (0.0–0.7)	2.4 ± 3.4 (1.7–3.0)	1 ± 1.5 (0.8–1.8)	0.6 ± 1.4 (0.1–1.0)	0.581
VAS pain (95% CI)	5 ± 3.2 (3.2–6.8)	2.8 ± 2.2 (2.0–3.6)	2.3 ± 2.1 (0.7–4.0)	6.4 ± 1.5 (4.6–8.2)	5 ± 2.1 (2.1–7.9)	4.5 ± 2.1 (2.8–6.2)	6.9 ± 2.2 (3.9–9,9)	4.8 ± 2.5 (1.0–8.6)	4.4 ± 2.4 (3.8–5.3)	0.900
LEI (95% CI)	2 ± 2.1 (0.1–2.9)	2.0 ± 2.5 (0.7–2.3)	3 ± 4.3 (0.2–5.8)	1.2 ± 0.8 (0.3–2.1)	0.2 ± 0.4 (0.0–0.4)	0.3 ± 0.5 (0.0–0.7)	1.0 ± 1.7 (0.6–1.4)	0.4 ± 0.8 (0.2–0.6)	0.4 ± 1.2 (0.0–0.9)	0.381
pGA (95% CI)	6.5 ± 2.6 (5.1–7.9)	2.2 ± 1.7 (0.7–3.7)	1.9 ± 1.8 (0.5–3.3)	6.6 ± 1.6 (4.7–8.5)	6.1± 1.8 (5.0–7.2)	6.4 ± 1.7 (4.7–8.1)	6.2 ± 2.2 (5.0–7.4)	4.4 ± 2.3 (2.8–6.0)	4.3 ± 2.1 (3.0–5.6)	0.004 *
phGA (95% CI)	4.6 ± 2.7 (1.5–7.7)	2 ± 1.5 (1.1–2.7)	1.3 ± 1.5 (0.1–2.4)	4.6 ± 1.8 (3.0–6.2)	3.2 ± 1.7 (0.4–6.0)	2.4 ± 1.9 (0.9–3.9)	5.1 ± 2.3 (4.3–5.9)	3.1 ± 2.1 (2.1–4.1)	2.3 ± 2.1 (1.5–2.8)	0.577
ESR mm/h (95% CI)	16.2 ± 9.5 (10.1–22.3)	11.6 ± 10.6 (7.0–16.0)	11.6 ± 9.7 (3.4–19.7)	21.2 ± 17.3 (14.4–28.0)	18.5 ± 11 (9.7–27.3)	12.3 ± 6.7 (6.7–18.0)	20.9 ± 14.5 (19.4–22.4)	18.8 ± 13 (16.7–20.9)	22.5 ± 18.5 (15.5–27.8)	0.202
CRP mg/L (95% CI)	4.1 ± 4.4 (0.3–7.9)	3.3 ± 2.1 (1.2–5.4)	3 ± 3.1 (0.6–5.5)	4.3 ± 6.8 (1.0–7.6)	0.8 ± 0.4 (0.1–1.5)	0.7 ± 0.4 (0.3–1.1)	3.5 ± 4.5 (2.7–4.3)	2.9 ± 4.2 (1.2–4.6)	3.1 ± 6.1 (1.1–4.8)	0.308
PASI (95% CI)	6 ± 4.5 (1.2–10.8)	0.9 ± 1.5 (0.0–1.8)	0.3 ± 0.6 (0.1–0.7)	4.3 ± 7.5 (1.2–7.4)	0.8 ± 1.8 (0.1–1.5)	0.6 ± 1.2 (0.0–1.3)	2.9 ± 3.6 (1.8–4.0)	0.5 ± 1.0 (0.2–0.8)	0.3 ± 0.8 (0.0–0.6)	0.134
HAQ (95% CI)	0.5 ± 0.4 (0.3–0.7)	0.4 ± 0.3 (0.2–0.7)	0.2 ± 0.3 (0.0–0.4)	0.2 ± 0.2 (0.0–0.4)	0.3 ± 0.2 (0.0–0.6)	0.2 ± 0.1 (0.0–0.4)	0.8 ± 0.4 (0.6–1.0)	0.6 ± 0.5 (0.4–0.8)	0.5 ± 0.4 (0.3–0.8)	0.686

Abbreviation: BMI: body mass index; CRP: C reactive protein; CI: confidence interval; csDMARDs: classic synthetic disease-modifying anti-rheumatic drugs; DAPSA: disease activity in psoriatic arthritis score; ESR: erythrocytes sedimentation rate; GC: glucocorticoid; HAQ: health assessment questionnaire; LEI: Leeds Enthesitis Index; PASI: Psoriasis Area and Severity Index; pGA: patient’s global assessment; phGA: physician global assessment; SJC: swollen joint count; TJC: tender joint count; VAS: Visual Analogue Scale. ° bDMARD-naive vs. ≥2 previous bDMARDs *p* = 0.016; 1 previous bDMARD vs. ≥2 previous bDMARDs *p* = 0.006. ^ bDMARD-naive vs. ≥ 2 previous bDMARDs *p* = 0.001; 1 previous bDMARD vs. ≥2 previous bDMARDs *p* = 0.001. * 12th month bDMARD-naive vs. 12th month 1 bDMARD NR *p* = 0.0001; 12th month bDMARD-naive vs. 12th month ≥ 2 bDMARDs NR *p* = 0.011; 12th month 1 bDMARDs NR vs. 12th month ≥ 2 bDMARDs NR *p* = 0.017.

**Table 4:** Univariate and multivariate binary logistic regression analysis for predictors of reaching MDA at the 12th month.

	Univariate		Multivariate	
	OR (95% CI)	*p*-Value	OR (95% CI)	*p*-Value
Smoke	0.6 (0.167–2.1)	0.435		
BMI	0.9 (0.8–1.1)	0.577		
Obesity	1.1 (0.6–2.2)			
PsA disease duration (years)	1.02 (0.9–1.09)	0.535		
Psoriasis	2.9 (0.2–35.3)	0.397		
Enthesitis	1.1 (0.3–4.0)	0.770		
Dactylitis	0.3 (0.08–1.6)	0.203		
bDMARD-naive	7.1 (1.4–34.2)	0.0014	7.9 (1.3–44.8)	0.019
Concomitant csDMARDs	0.917 (0.2–3.2)	0.894		
Concomitant oral GC	1.3 (0.3–4.6)	0.688		
Baseline pGA	1.3 (1–1.7)	0.0480	1.1 (1–1.5)	0.046
Baseline phGA	0.7 (0.5–1.0)	0.118		
Baseline DAPSA	0.9 (0.6–1.4)	0.718		
Baseline PASI	1.1 (1–1.4)	0.0310	0.9 (0.8–1.1)	0.631
Baseline HAQ	0.4 (0.05–3.2)	0.416		
Fibromyalgia	3.1 (0.037–2.7)	0.298		
Diabetes	1.4 (0.4–4.9)	0.550		

Abbreviation: BMI: body mass index; csDMARDs: classic synthetic disease-modifying anti-rheumatic drugs; DAPSA: disease activity in psoriatic arthritis score; GC: glucocorticoid; HAQ: health assessment questionnaire; PASI: Psoriasis Area and Severity Index; pGA: patient’s global assessment; phGA: physician global assessment.

## Data Availability

All data is presented in the manuscript.
